# Exploring pharmacological approaches for managing cytokine storm associated with pneumonia and acute respiratory distress syndrome in COVID-19 patients

**DOI:** 10.1186/s13054-020-03020-3

**Published:** 2020-06-11

**Authors:** Irma Convertino, Marco Tuccori, Sara Ferraro, Giulia Valdiserra, Emiliano Cappello, Daniele Focosi, Corrado Blandizzi

**Affiliations:** 1 Unit of Pharmacology and Pharmacovigilance, Department of Clinical and Experimental Medicine, University of Pisa, Pisa, Italy; 2 Unit of Adverse Drug Reactions Monitoring, Pisa University Hospital, Pisa, Italy; 3 North-Western Tuscany Blood Bank, Pisa University Hospital, Pisa, Italy

**Keywords:** Sars-CoV-2, COVID-19, Cytokines, Interleukin-6, Tumor necrosis factor, Janus kinases, Molecular targeted therapy

## Abstract

Sars-CoV-2 complications include pneumonia and acute respiratory distress syndrome (ARDS), which require intensive care unit admission. These conditions have rapidly overwhelmed healthcare systems, with detrimental effects on the quality of care and increased mortality. Social isolation strategies have been implemented worldwide with the aim of reducing hospital pressure. Among therapeutic strategies, the use of immunomodulating drugs, to improve prognosis, seems promising. Particularly, since pneumonia and ARDS are associated with a cytokine storm, drugs belonging to therapeutic classes as anti-IL-6, anti-TNF, and JAK inhibitors are currently studied. In this article, we discuss the potential advantages of the most promising pharmacological approaches.

## COVID-19 infection

The COVID-19 pandemic has rapidly brought down not only the public health but also the social issues and the global economy, owing to the uncertainties surrounding the characteristics of the virus, the lack of a vaccine, and the relatively scarce effectiveness of currently available antiviral treatments (i.e. RNA-dependent RNA polymerase inhibitors in combination with hydroxychloroquine in patients with severe pneumonia) [1, 2].

In symptomatic patients, the infection can evolve towards a severe inflammatory response, involving several organs with lung injury and bilateral interstitial pneumonia. In this setting, acute respiratory distress syndrome (ARDS) can occur, and intensive care unit (ICU) along with mechanical ventilation is required [3]. Worldwide, among the current primary needs of healthcare systems, there is the implementation of measures that can improve the sustainability of the number of patients requiring hospitalization and ICU admission. In this scenario, the use of existing therapeutic options, able to mitigate the disease severity, could contribute to achieve this goal. In the present article, we discuss the main drugs known to exert immunomodulatory effects by targeting the cytokine pathways that may represent promising strategies for the management of COVID-19-related ARDS and pneumonia.

## The cytokine storm

The majority of patients with COVID-19 infection shows alterations of white blood cell counts, in particular lymphocytopenia. In patients requiring ICU admission, an increase in neutrophil count, D-dimer, blood urea, and creatinine levels have been detected as well as more severe lymphocytopenia. This condition is defined as a “cytokine storm” and is associated with high circulating levels of interleukins (IL)-2, IL-6, IL-7, IL-10, granulocyte colony-stimulating factor (G-CSF), 10 kDa interferon-gamma-induced protein (IP-10), monocyte chemo-attractant protein-1 (MCP-1), macrophage inflammatory protein 1α (MIP-1α), and tumor necrosis factor (TNF) [4, 5]. In particular, in ARDS patients, a strong depletion of peripheral blood T cells, along with a decreased recruitment of lymphocytes and neutralizing antibodies and an increased production of cytokines, was detected in the lungs [6]. This network of pathogenic factors is thought to drive a severe immune-mediated interstitial pneumonia and a delayed pulmonary clearance of COVID-19 [3].

Current evidence supports a close relationship between cytokine storm and disease severity. Indeed, ICU patients displayed higher serum levels of cytokines (G-CSF, IP-10, MCP-1, MIP-1α, and TNF–α) than those not requiring ICU. For instance, IL-6 and TNF-α levels in ICU patients were significantly higher when compared with non-ICU ones [7]. Patients with fatal outcome had higher serum concentrations of IL-6 than those survived: the IL-6 median levels reported by Zou et al. were 11.00 pg/mL (IQR 7.50–14.40) and 6.30 pg/mL (IQR 5.00–7.90), respectively, *p* < 0.0001. Similar findings were showed by Ruan et al. 11.4 pg/mL (IQR 8.5) in dead patients versus 6.8 pg/mL (IQR 3.61) in discharged ones [8, 9]. Furthermore, a significantly close relationship between IL-6 levels in critical COVID-19 patients with fatal outcome (64.0 pg/ml, IQR 25.6–111.9) and RNAemia was found, in particular, the 83.3% of patients with IL-6 > 100 pg/ml had positive levels of RNAemia, *r* 0.902. This suggests that high serum IL-6 along with RNAemia could be predictors of mortality [10]. Additionally, not only critically ill patients with ARDS have been associated with high cytokine serum levels but also non-severe patients with COVID-19. Indeed, IL-6 median serum levels were 36.10 pg/mL (IQR 23.00–59.20) in severe patients compared with 10.60 pg/mL (IQR 5.13–24.18) in those with mild disease, *p* 0.002 [11], and 6.69 pg/mL (IQR 4.44–12.43) in patients with SpO_2_ ≥ 90% in comparison with 51.69 pg/mL (IQR 34.31–161.65) in those with SpO_2_ < 90%, *p* < 0.001, as well as the TNF-α levels (2.08 pg/mL, IQR 1.93–2.35 even in the conditions with SpO_2_ ≥ 90%) [12]. These data were confirmed by Qin et al.; IL-6 median serum levels in severe and non-severe patients were 25.2 pg/mL (IQR 9.5–54.5) and 13.3 pg/mL (IQR 3.9–41.1), respectively; and TNF-α median serum levels were 8.7 pg/mL (IQR 7.1–11.6) in severe patients and 8.4 pg/mL (IQR 6.9–10.4) in non-severe ones [6].

Based on this knowledge, it has been proposed that the modulation of the above cytokines could represent an interesting approach to improve the prognosis of patients with COVID-19 pulmonary complications, both pneumonia and ARDS. Recently, The Food and Drug Administration has allowed the emergency use of a device aiming at purifying blood of ICU patients from the cytokine storm [13].

## Potential therapeutic target drugs

**Fig. 1:**
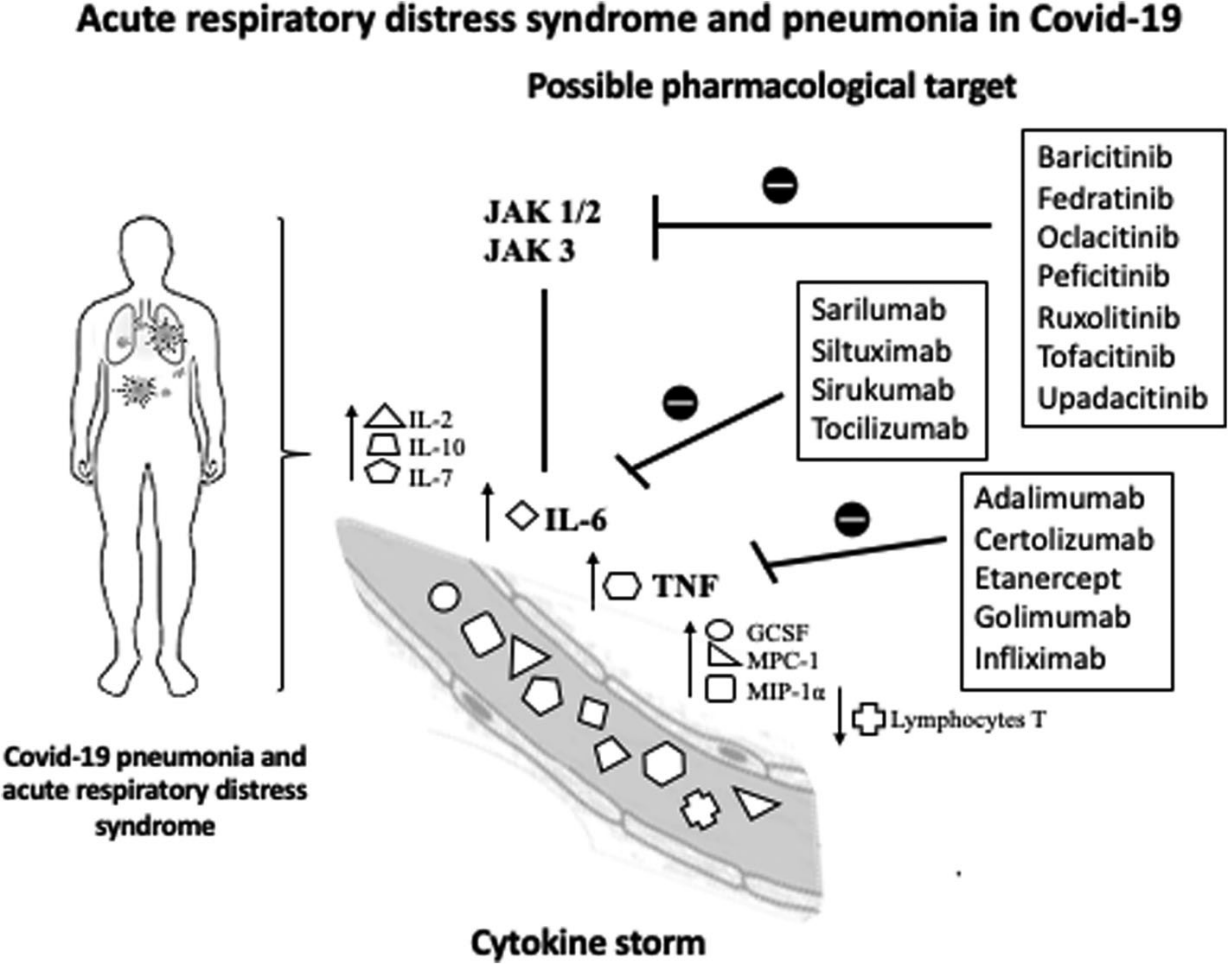
Cytokine storm and potential pharmacological targets in COVID-19-related ARDS and pneumonia. IL, interleukin; TNF, tumor necrosis factor, GCSF, granulocyte colony-stimulating factor; JAK, Janus kinase; MCP, monocyte chemoactractant protein; MIP, macrophage inflammatory protein

Several drugs, endowed with modulating activity on cytokine pathways, including anti-IL-6, anti-TNF, and Janus kinase (JAK) inhibitors, currently approved for the treatment of immune-mediated inflammatory diseases, have been suggested or could be yet taken into account for experimental use in COVID-19 patients with ARDS and/or pneumonia (Fig. 1).

### Anti-IL-6

Tocilizumab is a humanized, immunoglobulin G1κ (IgG1κ) anti-human IL-6 receptor (IL-6R) monoclonal antibody approved for some immune-mediated inflammatory rheumatic diseases. Clinical evidence supports the view that this drug is an effective therapeutic option, with a good risk-benefit profile, in cytokine storm syndromes [14]. In China, its off label use has been tested in 21 ICU ARDS patients with favorable results after 24–48 h in 20/21 patients [15]. Moreover, a multicenter randomized clinical trial in COVID-19 patients with ARDS, treated with tocilizumab at a dose of 4 ~ 8 mg/kg once, and an additional same dose when fever persists within 24 h after the first administration, has been approved in China [16]. The Italian Medicine Agency has recently authorized a trial on the use of tocilizumab in COVID-19 patients with ARDS [17]. This initiative was pushed on also by promising results published on Italian newspapers. Particularly, some patients treated with tocilizumab at the “Pascale” Cancer Institute in Naples showed disease improvements within 24 h and one of them did not require mechanical ventilation 2 days after starting tocilizumab [15].

Another monoclonal antibody belonging to anti-IL-6 drug class, siltuximab, currently approved in multicentric Castleman disease with HIV-negative and human herpesvirus-8 negative, is under investigation for ARDS in COVID-19 patients. In particular, an observational case-control study evaluating siltuximab in ICU patients with ARDS-related COVID-19 is performing at Papa Giovanni XXIII hospital in Bergamo, Italy [18]. Preliminary results have shown promising outcomes as the clinical improvement in the 33% of treated ICU patients [19]. In addition, a multicenter open-label randomized clinical trial is studying the benefit risk profile of siltuximab, as a single therapeutic option or in combination with anakinra, at a single dose of 11 mg/kg, in comparison with tocilizumab or anakinra, alone or in combination, in ARDS patients with COVID-19 [20]. Evidence suggested a higher binding affinity to IL-6 involving siltuximab than tocilizumab but less insights are currently available on the effects of siltuximab in cytokine storm [21].

Based on the results expected with tocilizumab and siltuximab, other anti-IL-6 drugs, currently approved for rheumatoid arthritis, namely sarilumab and sirukumab, could be studied in ARDS and pneumonia patients with COVID-19. Notably, sarilumab has higher affinity for its target and a longer half-life than tocilizumab; thus, a sustained therapeutic effect could be achieved by administration of only one single dose [22, 23]. On March 19^th^, 2020, a clinical trial evaluating the efficacy and safety of high dose and low dose of sarilumab in COVID-19 patients was started [24]. Subsequently, further clinical trials have followed, investigating the benefit risk profile of sarilumab in patients with COVID-19-related ARDS, at a dose of 200 mg or 400 mg, as single or repeated administration, subcutaneously or intravenously [25–28].

Sirukumab neutralizes IL-6 specifically and directly by preventing its binding to its membrane receptor [29], and thus, it leads to a subsequent suppression of IL-6 biological actions. In a phase I trial, sirukumab showed linear pharmacokinetics with long half-life, low immunogenicity, and a good safety profile [30]. Accordingly, it could represent a promising pharmacological option for counteracting the high IL-6 levels occurring in ARDS patients.

### Anti-TNF

Anti-TNF drugs, including infliximab, adalimumab, etanercept, golimumab, and certolizumab, could be tested also for COVID-19-related ARDS and pneumonia. In China, a clinical trial on adalimumab in COVID-19 patients was recently approved [31]. Infliximab, adalimumab, and golimumab are IgG_1_ monoclonal antibodies, while etanercept is a fusion protein of two human TNF type 2 receptor moieties linked with the Fc region of a human immunoglobulin, and certolizumab is the pegylated Fab domain obtained from a humanized anti-TNF IgG monoclonal antibody [32]. Differences in the inhibitory mechanism were shown among these drugs, due to their different molecular binding patterns with TNF sites [33]. All anti-TNF drugs display higher binding affinity to soluble TNF than its membrane-bound form, with golimumab and etanercept showing the highest level [34]. Greater binding avidity to soluble TNF was reported for etanercept than infliximab and adalimumab [35]. Heterogeneity was also found in the neutralizing activity of anti-TNF drugs to soluble TNF, while that to transmembrane TNF was comparable [34]. Infliximab and adalimumab displayed a greater binding activity for FcγRII and FcγRIII than etanercept, but the latter was able to bind FcγRI with higher affinity [36]. FcγRs play important roles in the modulation of immune responses, which rely on cytokines and vasoactive mediators [37]. In addition, a review showed that the proteins coded by the virus alter the complement system control and thus contribute to lung viral damages [3]. Out of the anti-TNF drugs, the IgG_1_ monoclonal antibodies have a complement-dependent cytotoxicity activity [38] that could be explored in the COVID-19 infection. The known differences in pharmacokinetics and pharmacodynamics among anti-TNF drugs support the need for testing these agents in COVID-19-related ARDS and pneumonia patients without particular priorities, in order to identify the best option. Other selection criteria, including the administration route, the possible positive or negative interactions resulting from combination with other drugs (i.e., hydroxychloroquine) and the costs (i.e., the use of biosimilar anti-TNF available) should be considered.

### JAK inhibitors

Anti-JAK drugs (such as ruxolitinib, tofacitinib, baricitinib, oclacitinib, fedratinib, upadacitinib, and peficitinib) [39] should be considered also among the options for clinical investigations in COVID-19-related ARDS and pneumonia patients. JAKs are involved in JAK/STAT signaling associated with the receptors of a large variety of cytokines. In particular, STAT-1 and STAT-3 pathways are activated by binding of IL-6 to its receptor (IL-6R) [40]. Tofacitinib acts as a non-selective inhibitor of all known JAKs (JAK1, JAK2, JAK3, TYK2) with moderate specificity for JAK1 and JAK3. Baricitinib inhibits selectively JAK1 and JAK2 [41]. Both are approved by the European Medicines Agency (EMA); baricitinib for rheumatoid arthritis and tofacitinib for both rheumatic disorders and ulcerative colitis. Ruxolitinib is a JAK1/JAK2 inhibitor approved by the Food and Drug Administration (FDA) for psoriasis, myelofibrosis, and rheumatoid arthritis. Upadacitinib (anti-JAK1), fedratinib (anti-JAK2), and oclacitinib (anti-JAK1) were approved by FDA for rheumatoid arthritis, myelofibrosis, and dermatitis, respectively [42]. Peficitinib (anti-JAK3) was approved for rheumatoid arthritis only in Japan [43].

Tofacitinib and upadacitinib showed potent inhibitory activities on JAK1/3-dependent cytokines, both pathways being involved in lymphocyte activation. Tofacitinib, baricitinib, and upadacitinib displayed also inhibitory actions on the JAK2/TYK2-dependent signaling of IL-3, GM-CSF, and G-CSF. Tofacitinib was shown to act as the most potent inhibitor of G-CSF (JAK2/TYK2). Moreover, tofacitinib, baricitinib, and upadacitinib inhibited IL-6 and interferon (IFN) γ (JAK1/2), as well as IL-10 and IFN-α (JAK1/TYK2), with tofacitinib appearing as the strongest inhibitor of IL-6, IFN-γ, and IL-10 signals [44]. Evidence suggests that baricitinib, fedratinib, and ruxolitinib are also inhibitors of numb-associated kinases (NAK), involved in viral endocytosis and replication. Baricitinib showed the highest affinity for AAK1 than ruxolitinib and fedratinib. Thus, besides exerting putative anti-inflammatory effects in ARDS patients, it is expected also to reduce viral infectivity [45, 46]. Of note, a clinical trial on such antiviral effect is going to start with ruxolitinib [47], and an open-label trial is evaluating its efficacy and safety at a dose of 10 mg twice a day in COVID-19 patients with ARDS [48]. Furthermore, an expanded access program of the 5 mg ruxolitinib formulation is ongoing in severe COVID-19 patients with ≥ 6 years old [49]. Finally, an open label clinical trial is evaluating the benefit risk profile of baricitinib at a dose of 2 mg a day for 10 days in moderate and severe adult COVID-19 patients [27]. Whenever JAK inhibitors could be identified as an effective pharmacological option in COVID-19-related ARDS and pneumonia, their cost and safety issues, particularly the risk of thromboembolic events for some of them, should be taken into account [50].

## Conclusions

Several drugs targeting cytokine pathways hold the potential for providing benefits in COVID-19-related ARDS and pneumonia. Anti-IL-6, anti-TNF, and anti-JAK medications, currently available for treating other immune-dependent inflammatory diseases, are expected to exert favorable effects in this setting, based on their mechanism of action. In the current emergency situation and without reliable therapeutic options, their benefit risk-profile will likely be favorable. Cost and safety will be a priority at a later phase of the epidemic, when more robust data will be available.

## Data Availability

Not applicable.

## Abbreviations

ARDS: Acute respiratory distress syndrome
EMA: European Medicines Agency
FDA: Food and Drug Administration
G-CSF: Granulocyte colony-stimulating factor
ICU: Intensive care unit
IFN: Interferon
IgG_1_κ: Immunoglobulin G_1_κ
IL: Interleukins
IL-6R: Interleukin-6 receptor
JAK: Janus kinase
IP-10: 10 kDa interferon-gamma-induced protein
MCP-1: Monocyte chemo-attractant protein-1
MIP-1α: Macrophage inflammatory protein 1α
RNA: Ribonucleic acid
TNF: Tumor necrosis factor
